# Low microbial diversity, yeast prevalence, and nematode-trapping fungal presence in fungal colonization and leaf microbiome of *Serjania erecta*

**DOI:** 10.1038/s41598-024-66161-3

**Published:** 2024-07-04

**Authors:** Samylla Tássia Ferreira de Freitas, Fabiano Guimarães Silva, Layara Alexandre Bessa, Ueric José Borges de Souza, Damiana Souza Santos Augusto, Giselle Santos de Faria, Luciana Cristina Vitorino

**Affiliations:** 1 Laboratory of Agricultural Microbiology, Instituto Federal Goiano – campus Rio Verde, Highway Sul Goiana, Km 01, Rio Verde, GO 75901-970 Brazil; 2 Laboratory of Plant Mineral Nutrition, Instituto Federal Goiano, campus Rio Verde, Rio Verde, Brazil; 3 Bioinformatics and Biotechnology Laboratory, Federal University of Tocantins, Campus of Gurupi, Gurupi, TO 77410-570 Brazil

**Keywords:** Cerrado plant microbiome, Endophytic fungi, Epiphytic fungi, Nematode-trapping fungi, Metagenomic study, Plant symbiosis, Microbial communities

## Abstract

Medicinal plant microbiomes undergo selection due to secondary metabolite presence. Resident endophytic/epiphytic microorganisms directly influence plant’s bioactive compound synthesis. Hypothesizing low microbial diversity in *Serjania erecta* leaves, we assessed leaf colonization by epiphytic and endophytic fungi. Given its traditional medicinal importance, we estimated diversity in the endophytic fungal microbiome. Analyses included scanning electron microscopy (SEM), isolation of cultivable species, and metagenomics. Epiphytic fungi interacted with *S. erecta* leaf tissues, horizontally transmitted via stomata/trichome bases, expressing traits for nematode trapping. Cultivable endophytic fungi, known for phytopathogenic habits, didn’t induce dysbiosis symptoms. This study confirms low leaf microbiome diversity in *S. erecta*, with a tendency towards more fungal species, likely due to antibacterial secondary metabolite selection. The classification of *Halicephalobus* sp. sequence corroborated the presence of nematode eggs on the epidermal surface of *S. erecta* by SEM. In addition, we confirmed the presence of methanogenic archaea and a considerable number of methanotrophs of the genus *Methylobacterium.* The metagenomic study of endophytic fungi highlighted plant growth-promoting yeasts, mainly *Malassezia*, *Leucosporidium*, *Meyerozyma*, and *Hannaella*. Studying endophytic fungi and *S. erecta* microbiomes can elucidate their impact on beneficial bioactive compound production, on the other hand, it is possible that the bioactive compounds produced by this plant can recruit specific microorganisms, impacting the biological system.

## Introduction

Plant resident microbiota comprises epiphytic and endophytic fungi with important functional traits ranging from plant growth promotion to antibiosis to phytopathogens^1,2^. These fungi are non-pathogenic fungal representatives that grow in symbiotical association with plants^3–5^. Horizontal transmission from the epiphytic to the endophytic system and from the internal system to the plant surface^6^ has been demonstrated. This is because endophytes can emerge to sporulate during plant senescence or from host tissues^7^. However, each plant hosts epiphytic and endophytic microbiomes that are still unknown for most plant species. These microbiomes can be important sources of biotechnological solutions for agriculture and medicine due to their functional traits and bioactive compound synthesis^8,9^.

Some studies have associated the microbiome of medicinal plants with the ability of the plants to synthesize specific metabolites^10,11^. Studies examining endophytic fungi from *Stryphnodendron adstringens*, an endemic species of the Cerrado biome, reported that fungal isolates and bark extracts collected from the genus *Diaporthe*^12,13^ exhibit antimicrobial activity^14–16^ and cytotoxicity against human cancer cell lines^15,17^. The analyses of microbiomes in Chinese medicinal plants (*Ainsliaea henryi*, *Dioscorea opposita*, *Potentilla discolor*, *Stellera chamaejasme*, *Ophiopogon japonicus*, *Juncus effusus* var. *decipiens*, and *Rhizoma arisaematis*)^18,19^ showed that each of these plants hosted a specific community of actinomycetes with significantly high and diverse rhizospheric and endophytic Actinobacteria, presenting antimicrobial and antitumor properties^20^. Based on these data, we decided to study epiphytic and endophytic fungi associated with the medicinal plant *Serjania erecta* Radlk (Sapindales: Sapindaceae). The Sapindaceae family comprises many plants that produce latex containing saponins in their leaves, roots and/or seeds. Therefore, many species of this family are known for their importance in traditional medicine^21^. *S. erecta* is popularly known in Brazil as *cipó de cinco folhas*, a native plant to the Cerrado, with distribution restricted to areas of this biome, north of the Caatinga and south of the Amazonia^22^. This species has been reported in the literature for important biological effects such as anti-inflammatory activity^23^, gastroprotective action^24^, nematicidal effects^25^, and antimicrobial effects^26^. Ethanolic extract of *S. erecta* leaves and roots can inhibit a range of bacteria and the methanolic extract of the leaves also inhibits the growth and development of insect pests, such as *Chrysodeixis includens*^27,28^.

Medicinal plants have a vast microbiome; however, resident species can be affected or selected based on the presence of metabolites that may have bactericidal or fungicidal action^11^. Studies pinpointed that the microbiome can be influenced by a diverse set of molecules, including: coumarins, glucosinolates, benzoxazinoids, camalexin, and triterpenes^29,30^. Hence, as medicinal plants contain a range of leaf metabolites such as flavonoids, alkaloids, and essential oils^31^, we hypothesized low microbial diversity in *S. erecta* leaves. Freitas et al.^31^ reported that the internal tissues of *S. erecta* leaves can be colonized by the biotrophic fungi. But as this work focused on morphoanatomical aspects, our aimed to assess *S. erecta* leaf colonization by epiphytic and endophytic fungi. We also aimed to estimate the diversity of species forming the endophytic fungal microbiome of this species due to its relevance in traditional medicine.

Despite its great biotechnological potential, no studies have examined *S. erecta* resident epiphytic and endophytic microbiomes. Therefore, the objectives of this study were to assess the presence of fungi in leaf tissues by scanning electron microscopy (SEM) and identify leaf endophytic fungi using cultivable species isolation and metagenomic method. We aim to contribute to the increasing understanding of plant-microorganism interactions, particularly those involving medicinal species. We considered the perspective of Dantas et al.^15^, who stated that Cerrado plants constitute a repository of endophytic fungi with the potential to act as a source of new bioactive substances that can be used in several areas. Thus, it is imperative to study the symbiotic microbiome of medicinal species found in Cerrado.

## Results

### Colonization of epidermal structures by epiphytic fungi

**Figure 1:**
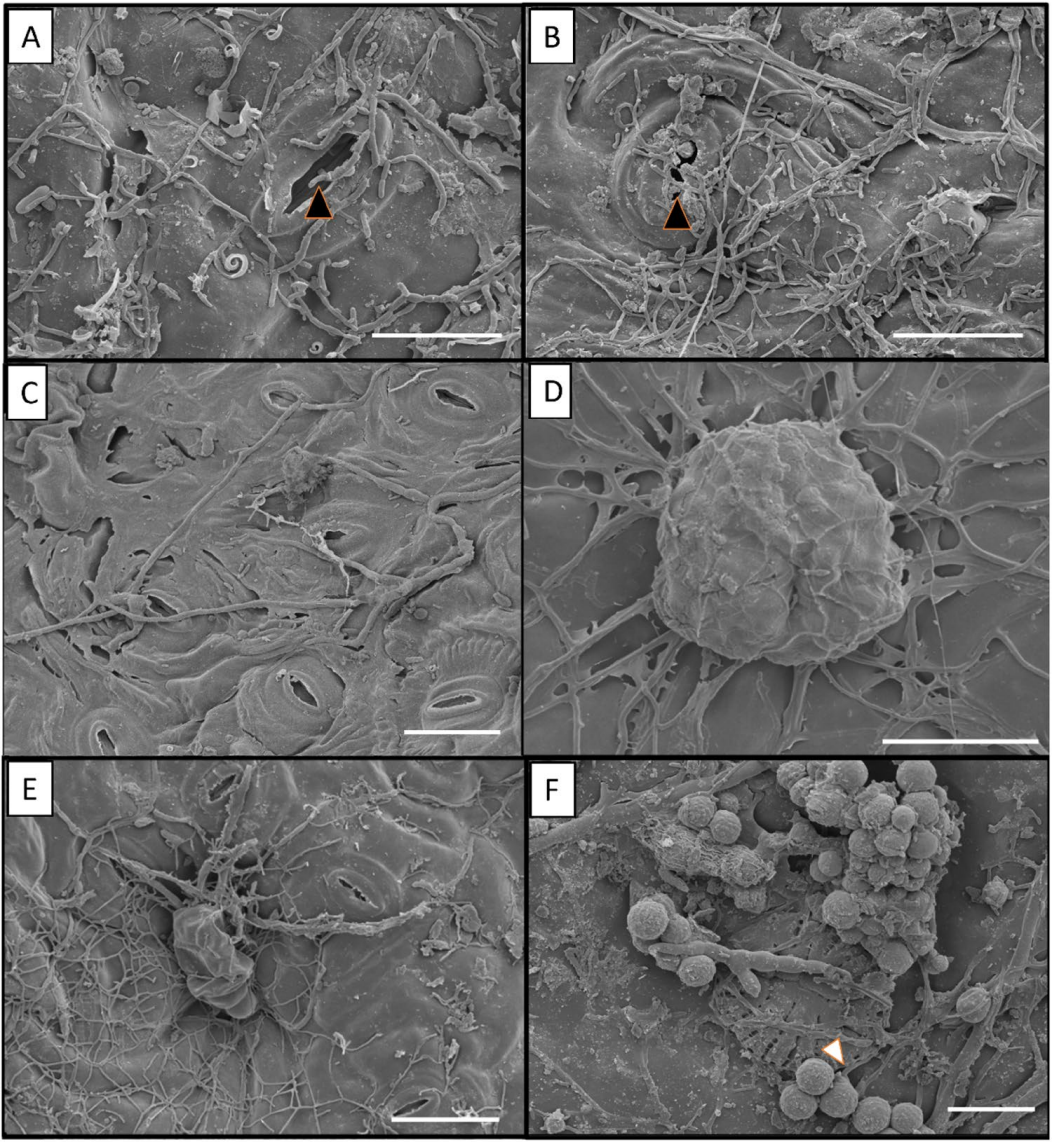
Colonization of *S. erecta* leaf surface by epiphytic fungi with penetration via the stomata and the base of the glandular and squamous trichomes located on the rachis (**A, B**), tooth (**C, D**), and blade (**E, F**). Black arrows indicate hypha penetration into the stomata, and white arrow indicates a spore noted during the germination process. A and B, 15 µm bar; C, E, and F, 12 µm bar; D, 25 µm bar.

Epiphytic fungi interact symbiotically with *S. erecta* leaf epidermis structures, exhibiting colonization via horizontal mechanisms, i.e., penetration through the stomata or the base of the glandular and squamous trichomes. This penetration was evidenced in these structures regardless of their location, i.e., in stomata and trichomes located on the rachis (Fig. 1A,B), tooth (Fig. 1C,D), and blade (Fig. 1E,F). We found a high level of hyphae colonization on the entire leaf surface, with the greatest accumulation of hyphae near stomata and trichomes. Spore accumulation was also observed on the blade and spores in the germination process (Fig. 1F).

**Figure 2:**
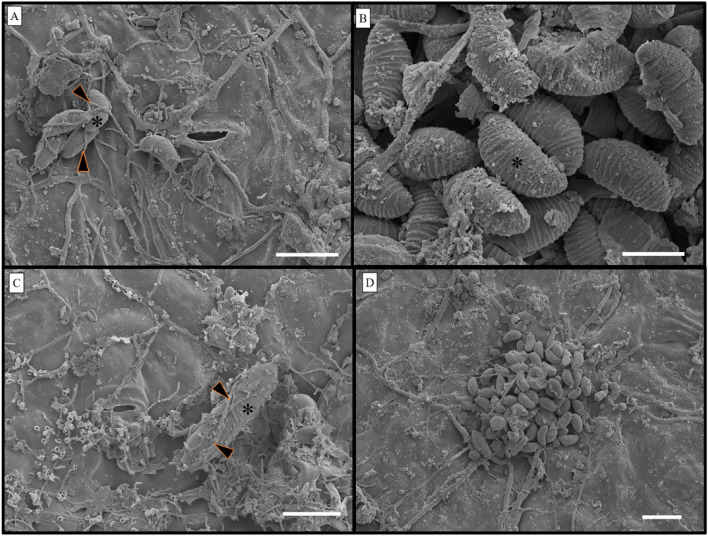
Colonization of *S. erecta* leaf surface by epiphytic fungi and nematodes; fungal hyphae adhered to the surface of the nematodes. * indicates nematodes, and arrows indicate hyphal adhesion and penetration through the eggshells. (**A**), 15 µm bars; (**B**), 6 µm bars; (**C**, **D**), 20 µm bars.

Surface electron micrographs of *S. erecta* epidermis also showed nematodes in the three leaf regions evaluated: rachis, tooth, and blade. However, fungal hyphae were always associated with nematodes (Fig. 2A–D), indicating that epiphytic fungi and nematodes compete to colonize leaf surface of *S. erecta*. Nematode eggs appear isolated (Fig. 2C) or in large groups (Fig. 2D). In this process, we saw fungi physically trapping nematodes (Fig. 2A,C) for parasitism or predation. This was evident from the presence of hyphae oriented perpendicularly to the eggshell, exerting a nematophagous effect. In addition, some eggs were damaged by the interaction with the hyphae (Fig. 2A,B).

### Colonization of leaf tissues by endophytic fungi

**Figure 3:**
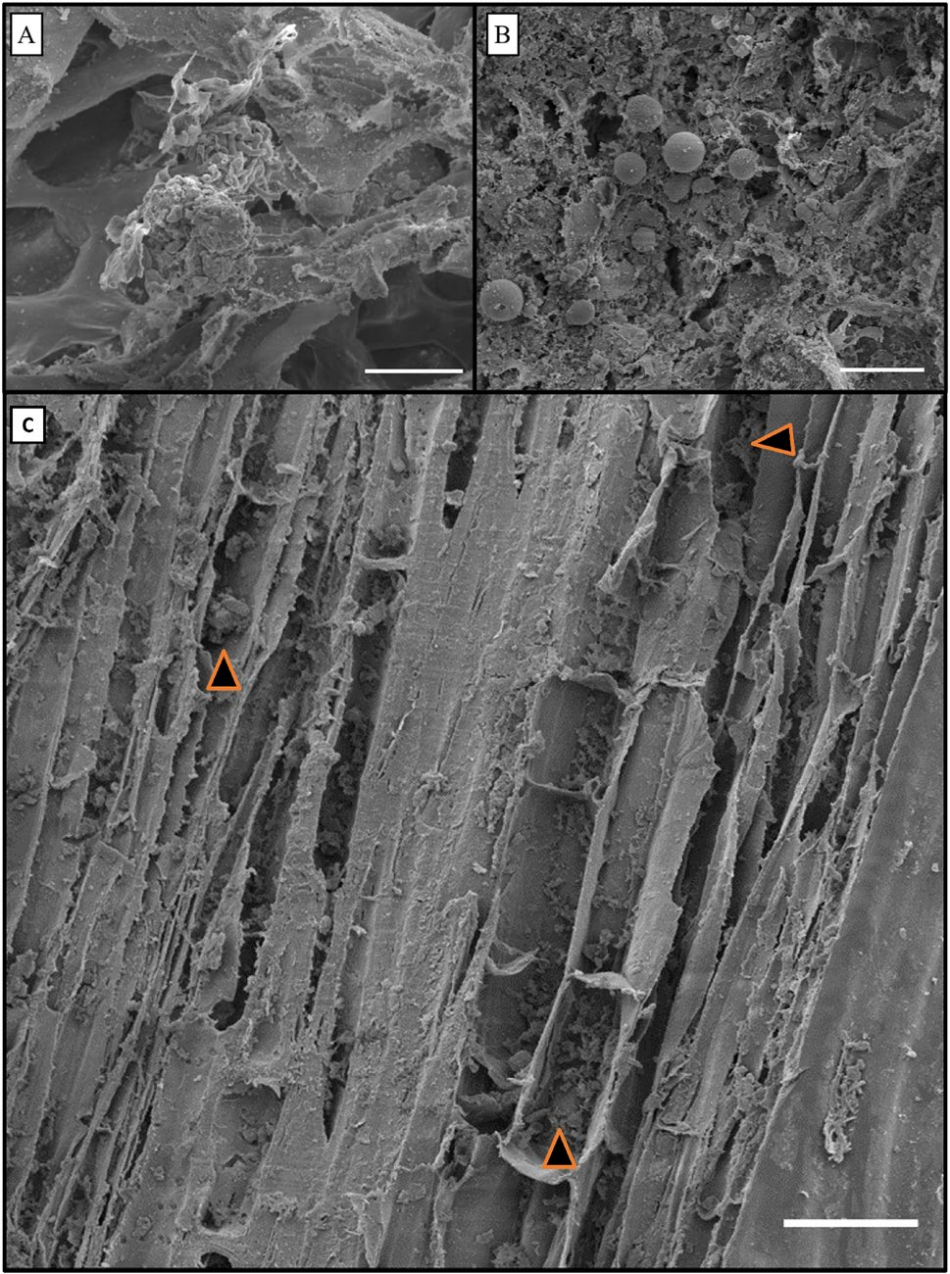
Paradermal section showing *S. erecta* leaf tissue colonization by endophytic fungi. Hyphae colonized internal tissues in the blade (**A**), and spores colonized glandular cavities in the teeth (**B**). Longitudinal section showing phloem colonization in the leaf rachis region; black arrows indicate the presence of fungal structures (**C**). A and B, 20 µm bar; C, 30 µm bar.

Paradermal sections of different *S. erecta* leaf regions showed endophytic fungi, with hyphae interacting with internal tissues in the rachis, tooth, and blade (Fig. 3A). Fungal spores were seen in glandular regions of the leaf teeth (Fig. 3B). Endophytic fungi were also seen in *S. erecta* conducting vessels, specifically in the phloem of primary and secondary veins in the leaf rachis (Fig. 3C), where microsclerocytes colonized the internal space of sieve tube elements.

### Isolation and identification of endophytic *fungi*

**Figure 4:**
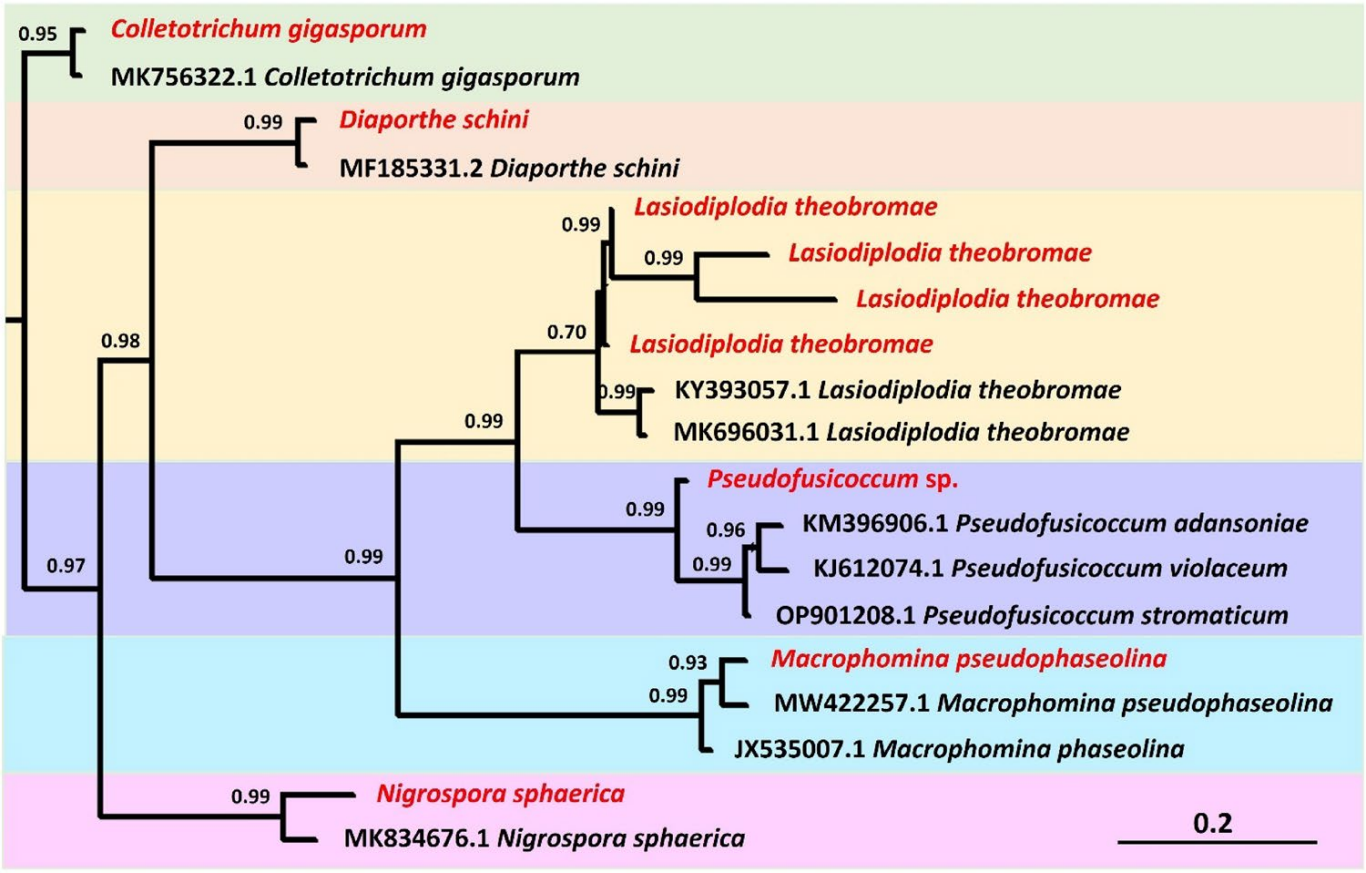
Similarity tree of endophytic fungi species isolated from *Serjania erecta* leaves. Phylogeny was recovered based on the internal transcribed spacer (ITS) and the *TUB2* gene. Numbers on the nodes represent the probability, and the bar at the bottom of the tree represents the genetic distance.

**Figure 5:**
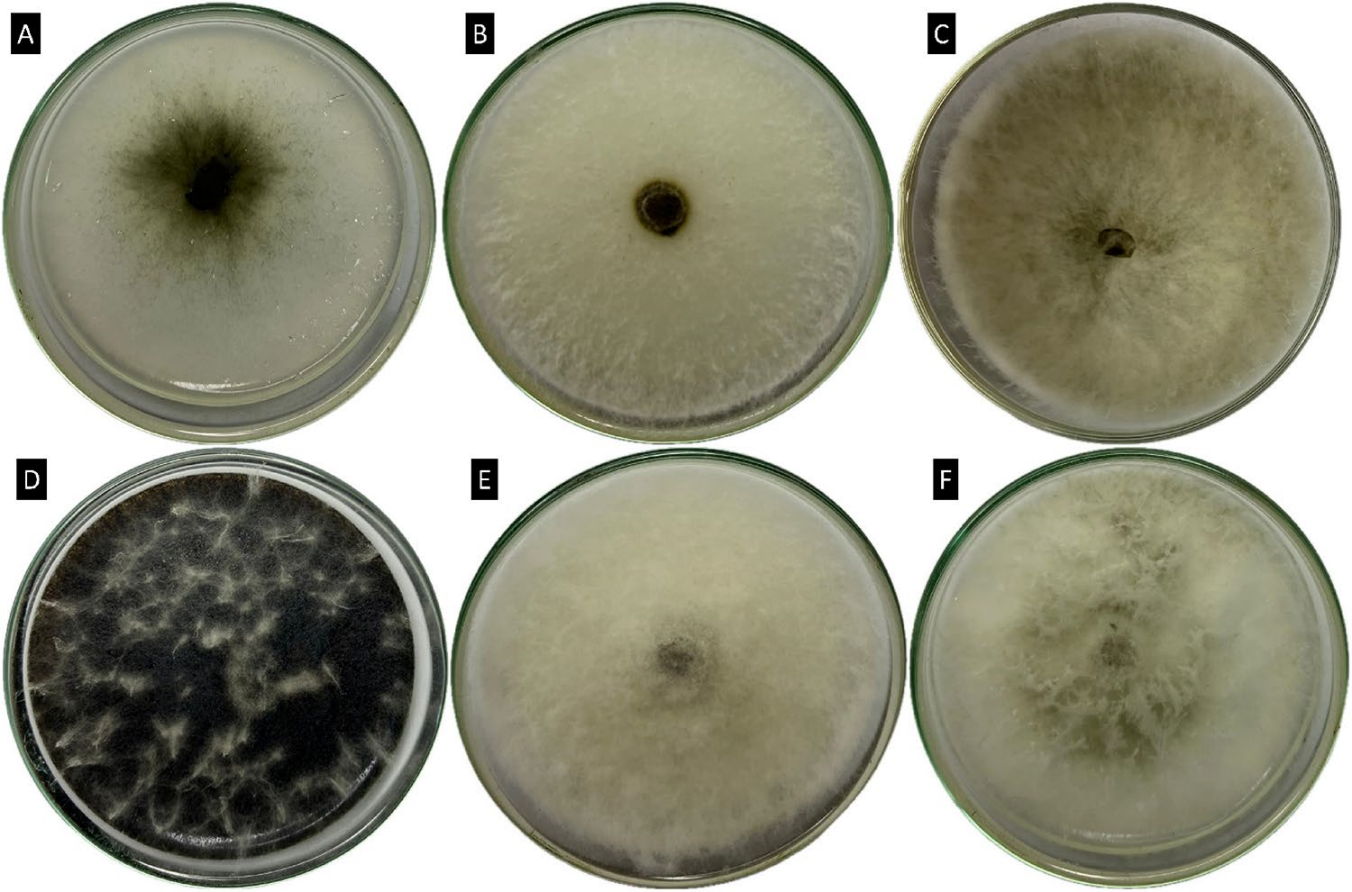
Endophytic fungi isolated from *S. erecta* leaves. *Colletotrichum gigasporum* (**A**), *Diaporthe schini* (**B**), *Lasiodiplodia theobromae* (**C**), *Macrophomina pseudophaseolina* (**D**), *Nigrospora sphaerica* (**E**), and *Pseudofusicoccum* sp. (**F**). Cultures observed at 7 days of growth on potato dextrose agar (PDA).

A total of nine fungal isolates of the phylum Ascomycota were obtained, and the following fungal species were identified: *Colletotrichum gigasporum* (one isolate), *Diaporthe schini* (one isolate), *Lasiodiplodia theobromae* (four isolates), *Macrophomina pseudophaseolina* (one isolate), and *Nigrospora sphaerica* (one isolate) (Figs. 4, 5) (Genbank accesses described in Table 1S). An isolate from the genus *Pseudofusicoccum* was also found. Although characterization of the species was not possible, this isolate formed a stable and highly sustained cluster with *Pseudofusicoccum adansoniae, Pseudofusicoccum violaceum,* and* Pseudofusicoccum stromaticum.*

### Metagenomics of leaf endophytic colonization

The metagenome sequencing of three *S. erecta* samples generated > 1,081 million paired reads or > 332 Mbp of sequence data. After trimming and filtering, > 1,014 million paired reads remained in the dataset with an average of > 338,000 paired reads per sample. Thus, an average of 6.19% of the reads were excluded (Figure 1S). Quality control resulted in high-quality raw data, with Phred scores ranging from 30–40. The FastQC software showed alterations attributed to the removal of adapters, identifying a low percentage of unique reads, which indicated that these species are commonly found in the samples (Figure 2S). The percentage of single reads was higher in SE-1, followed by SE-3 and SE-2. The rarefaction curve showed that the Shannon diversity index, which quantifies species diversity, reached a plateau at a sequencing depth below 10,000 (Figure 3S). This plateau suggests that the sequencing process effectively captured the full extent of species richness in the samples analyzed. SE-1 had a higher Shannon diversity index than SE-3, and SE-3 had a higher index than SE-2.

**Figure 6:**
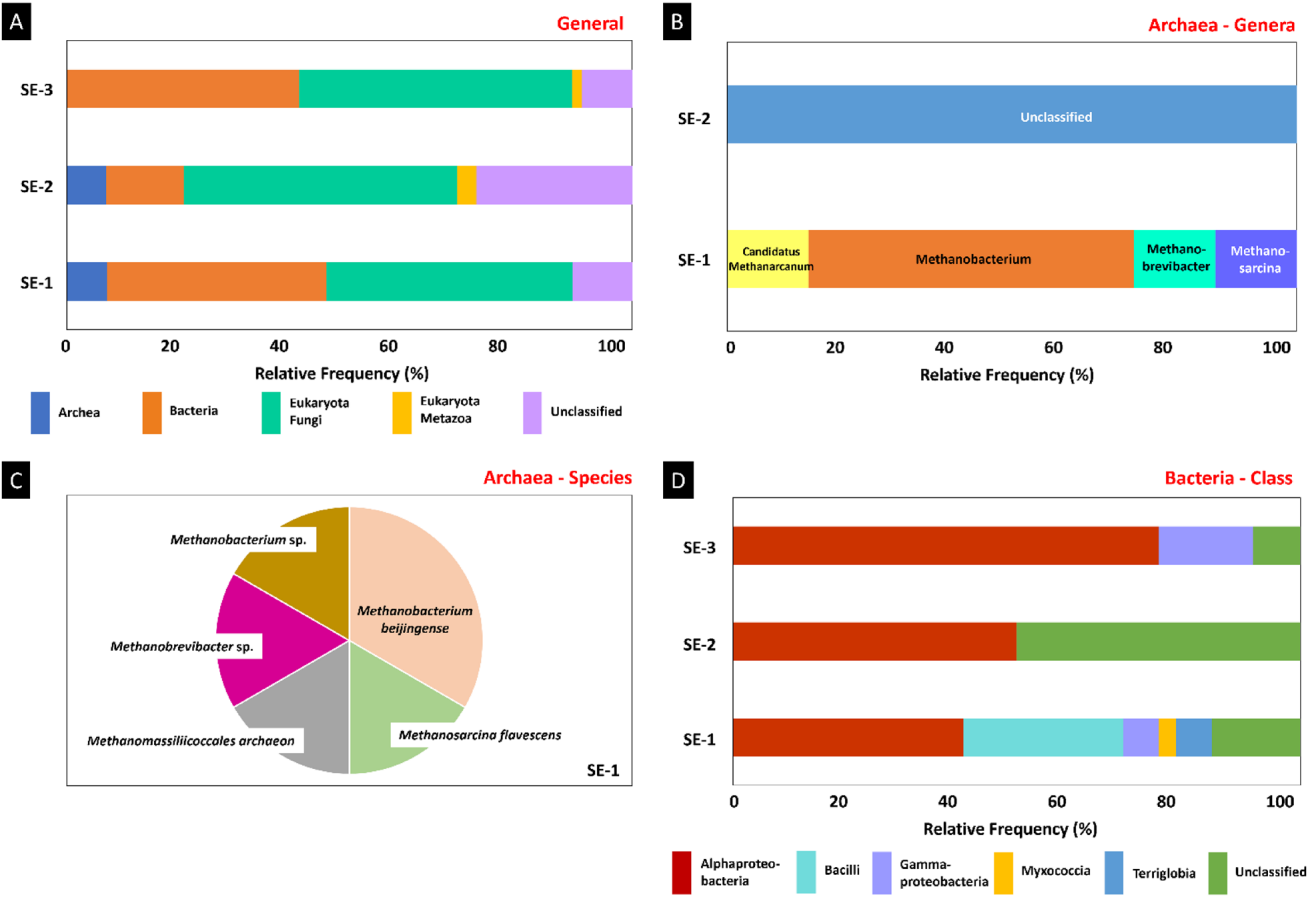
Metagenomic study of the endophytic colonization of *S. erecta* leaves. The three samples (SE-1, SE-2 and SE-3) were analyzed for the relative frequency of sequences attributed to organisms from different known domains (**A**), genus *Archaea* (**B**), *Archaea* species (**C**), and bacterial classes (**D**).

A total of 150 ASVs were identified from the 16S and 18S sequencing reads (data deposited in the SRA database, accession PRJNA1110285 and BioSample, accession SAMN41342363, SAMN41342364, SAMN41342365). These ASVs were classified into 90 species. There was a tendency toward a higher number of fungal species (43.53, 48.22, and 48.21% of the sequences observed in SE-1, SE-2, and SE-3, respectively) (Fig. 6A). Bacteria were observed at a lower frequency than fungi, with 38.82, 13.79, and 41.07% of sequences being found in SE-1, SE-2, and SE-3. Archaea were found in low percentages in SE-1 and SE-2 sequences (7.06 and 6.90%, respectively). Metazoan eukaryotes were seen in SE-2 and SE-3 (3.45 and 1.79%, respectively); however, all the sequences showed nematodes of the class Chromadorea, classified as *Halicephalobus* sp. (Table 2S). The percentage of novelty was higher in SE-2, with 27.59% of the sequences classified as not belonging to any of the known domains. This percentage was 10.59 and 8.93% in SE-1 and SE-3, respectively.

Most archaea sequences observed in SE-1 were assigned to the genus *Methanobacterium* (57.14%), with all the genera and species related to methanogenesis (Fig. 6B,C). All archaea obtained in SE-2 were assigned to unknown organisms belonging to these domains. SE-1 showed relatively more diverse bacterial classes compared to the other samples, but regardless of the sample, the class Alphaproteobacteria predominated (40.63, 50, and 75.00% of the bacteria in SE-1, SE-2, and SE-3, respectively) (Fig. 6D). Sequences from SE-1 and SE-3 were assigned to class Gammaproteobacteria (6.25 and 16.67%, respectively), and the percentage of novelty was high in SE-2 sequences (50%).

**Figure 7:**
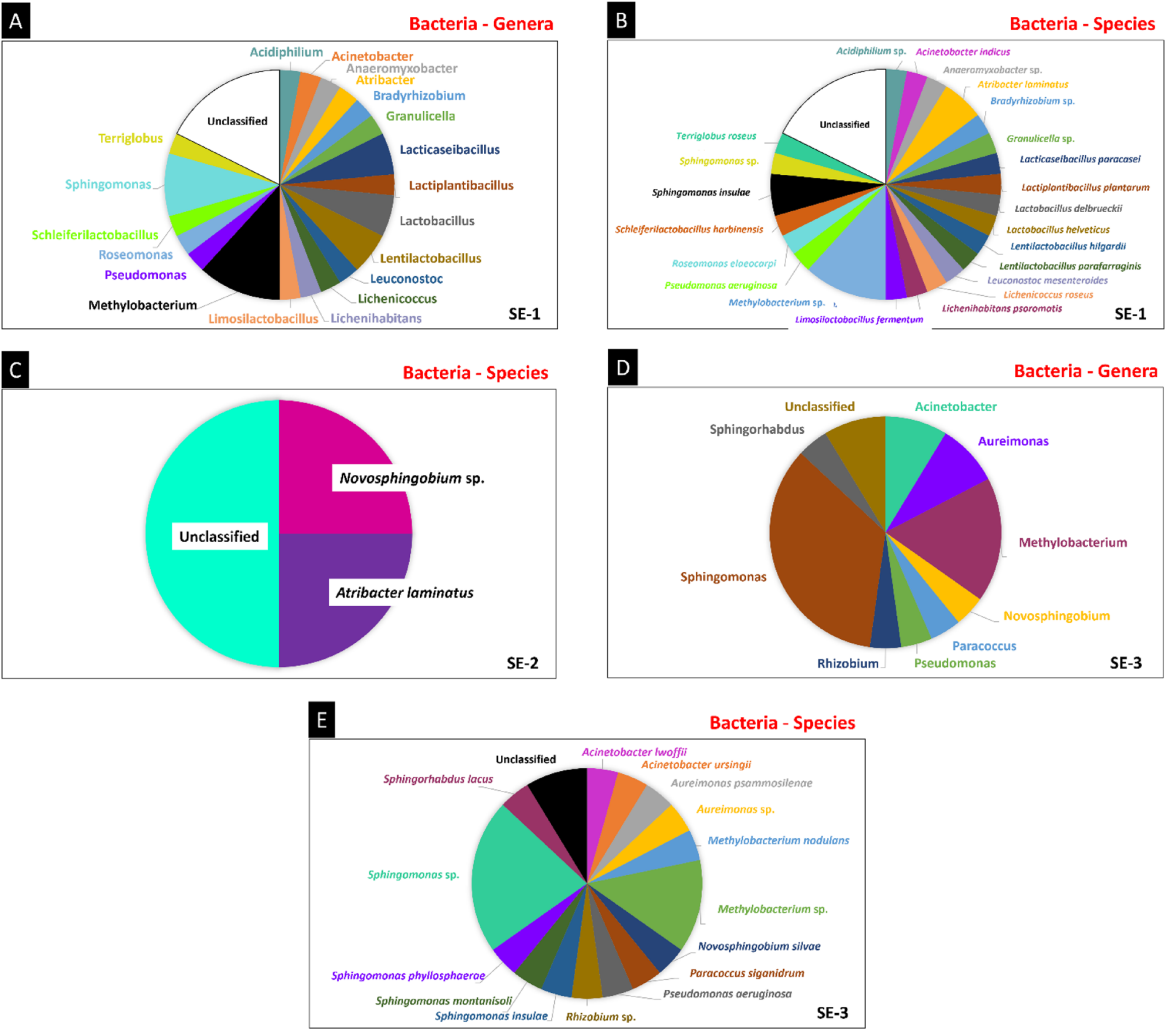
Metagenomic study of the endophytic colonization of *S. erecta* leaves. The three samples (SE-1, SE-2 and SE-3) were analyzed for the relative frequency of sequences attributed to bacterial genera and species in SE-1 (**A**, **B**), SE-2 (**C**), and SE-3 (**D**, **E**).

Most bacterial sequences in SE-1 could not be assigned to known bacterial genera or species (17.65%) (Fig. 7A,B). The genera *Methylobacterium* and *Sphingomonas* were the most frequent (11.76 and 8.92%), followed by *Lacticaseibacillus, Lactobacillus*, and *Lentilactobacillus* (5.88% each). Sequences of the genus *Methylobacterium* were classified as *Methylobacterium* sp. *Sphingomonas* were classified as *Sphingomonas insulae* and *Sphingomonas* sp. (5.98 and 2.94%, respectively). *Lacticaseibacillus* were classified as *Lacticaseibacillus paracasei* and *Lactiplantibacillus plantarum, Lactobacillus* as *Lactobacillus delbrueckii* and *Lactobacillus helveticus*, and *Lentilactobacillus* as *Lentilactobacillus hilgardii* and *Lentilactobacillus parafarraginis*.

The microbiome of SE-2 was less biodiverse, with 50% of the bacteria classified as unknown, 25% classified as *Novosphingobium* sp., and 25% as *Atribacter laminatus* (Fig. 7C). Most sequences in SE-3 were assigned to the genera *Sphingomonas* and *Methylobacterium* (34.79 and 17.75%, respectively), followed by *Acinetobacter* and *Aureimonas* (8.70% each) (Fig. 7D). Most *Methylobacterium* sequences were classified as *Methylobacterium* sp. (13.40%), but *Methylobacterium nodulans* sequences were also found (4.35%) (Fig. 7E). *Sphingomonas* sequences were classified as *Sphingomonas* sp. (21.74%), *Sphingomonas insulae* (4.35%), *Sphingomonas montanisoli* (4.35%), and *Sphingomonas phyllosphaerae* (4.35%). *Acinetobacter* species were classified as *Acinetobacter lwoffii* and *Acinetobacter ursingii* (4.35% each), *Aureimonas* as *Aureimonas psammosilene* and *Aureimonas* sp. (4.35% each).

SE-1 contained sequences of the species *Atribacter laminatus*, while SE-2 and SE-3 harbored bacteria of the genus *Novosphingobium*. Additionally, SE-1 and SE-3 exhibited the presence of the genera *Methylobacterium* and *Sphingomonas*, with the presence of the species *Sphingomonas insulae* being particularly noteworthy in both samples.

**Figure 8:**
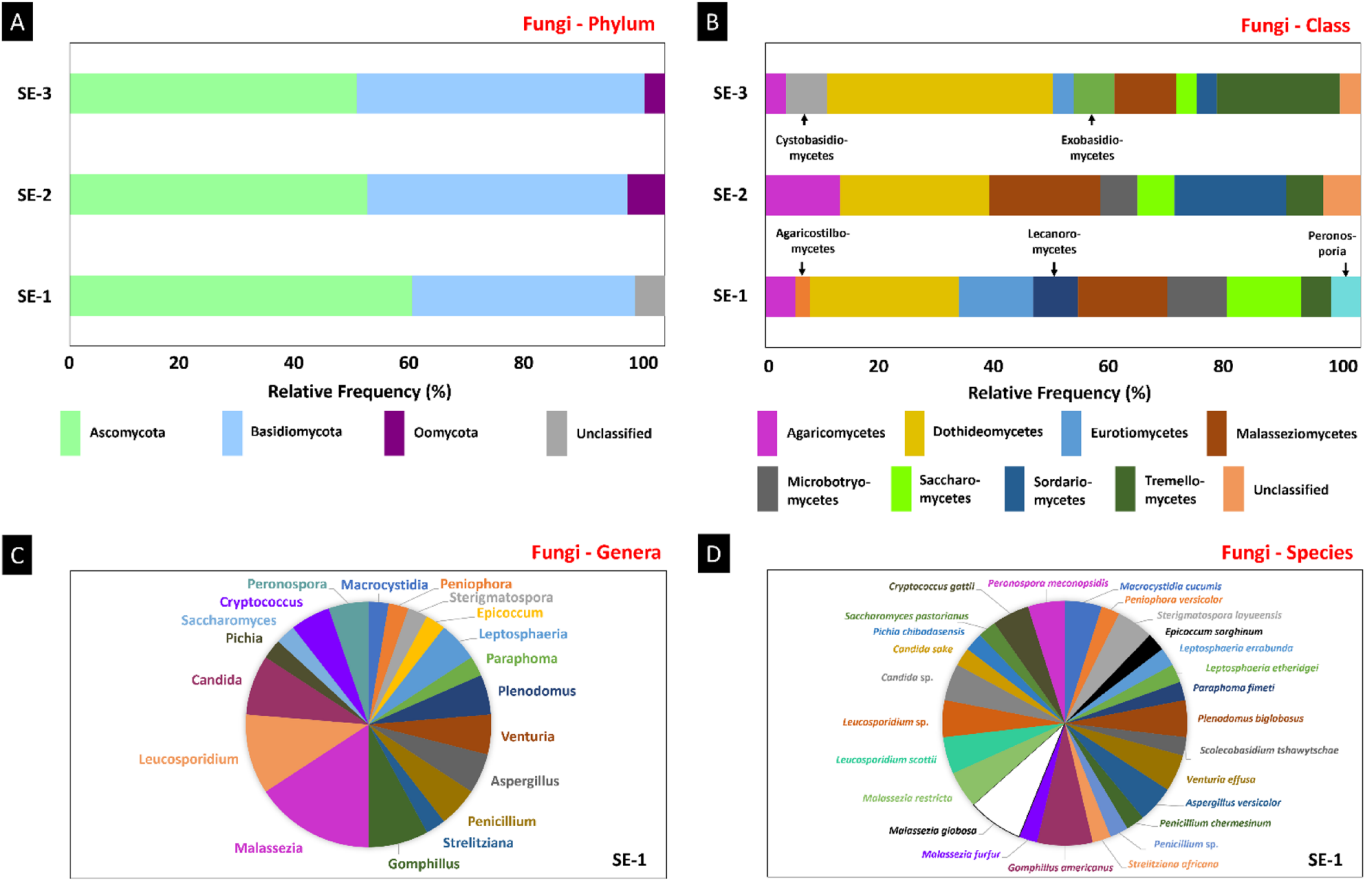
Metagenomic study of the endophytic colonization of *S. erecta* leaves. The three samples (SE-1, SE-2 and SE-3) were analyzed for the relative frequency of sequences attributed to fungal phyla (**A**) and classes (**B**). Fungal genera (**C**) and species (**D**) were analyzed in SE-1.

Sequences of endophytes of two fungal phyla were found in *S. erecta* SE-1 and SE-1 (Ascomycota and Basidiomycota). On the other hand, the samples also demonstrated sequences belonging to fungi not classified within the known phyla (3.45%) (Fig. 8A). Ascomycetes were the most frequent fungi, with sequences present in 57.50, 50.00, and 48.28% of the sequences classified in SE-1, SE-2, and SE-3, respectively. The fungal class Dothideomycetes was the most frequently found in the samples (25.00, 25.00, and 37.93% of sequences in SE-1, SE-2, and SE-3, respectively), followed by the classes Malasseziomycetes (15.00, 18.75, and 10.34%), Saccharomycetes (12.50, 6.25, and 3.45%), and Tremellomycetes (5.00, 6.25, and 20.69%) (Fig. 8B).

Most fungal sequences in SE-1 were assigned to the genera *Malassezia* (15.79%), *Leucosporidium* (10.53%), *Gomphillus* (7.89%), and *Candida* (7.89%) (Fig. 8C). The genus *Malassezia* included *Malassezia globosa* (7.84%), *Malassezia restricta* (5.20%), and *Malassezia furfur* (2.75%), and the genus *Leucosporidium* included *Leucosporidium scottii* (5.26%) and *Leucosporidium* sp. (5.26%). The genus *Gomphillus* included only one species, *Gomphillus americanos* (7.89%), and the genus *Candida* included *Candida sake* (5.26%) and *Candida* sp. (2.63%) (Fig. 8D).

**Figure 9:**
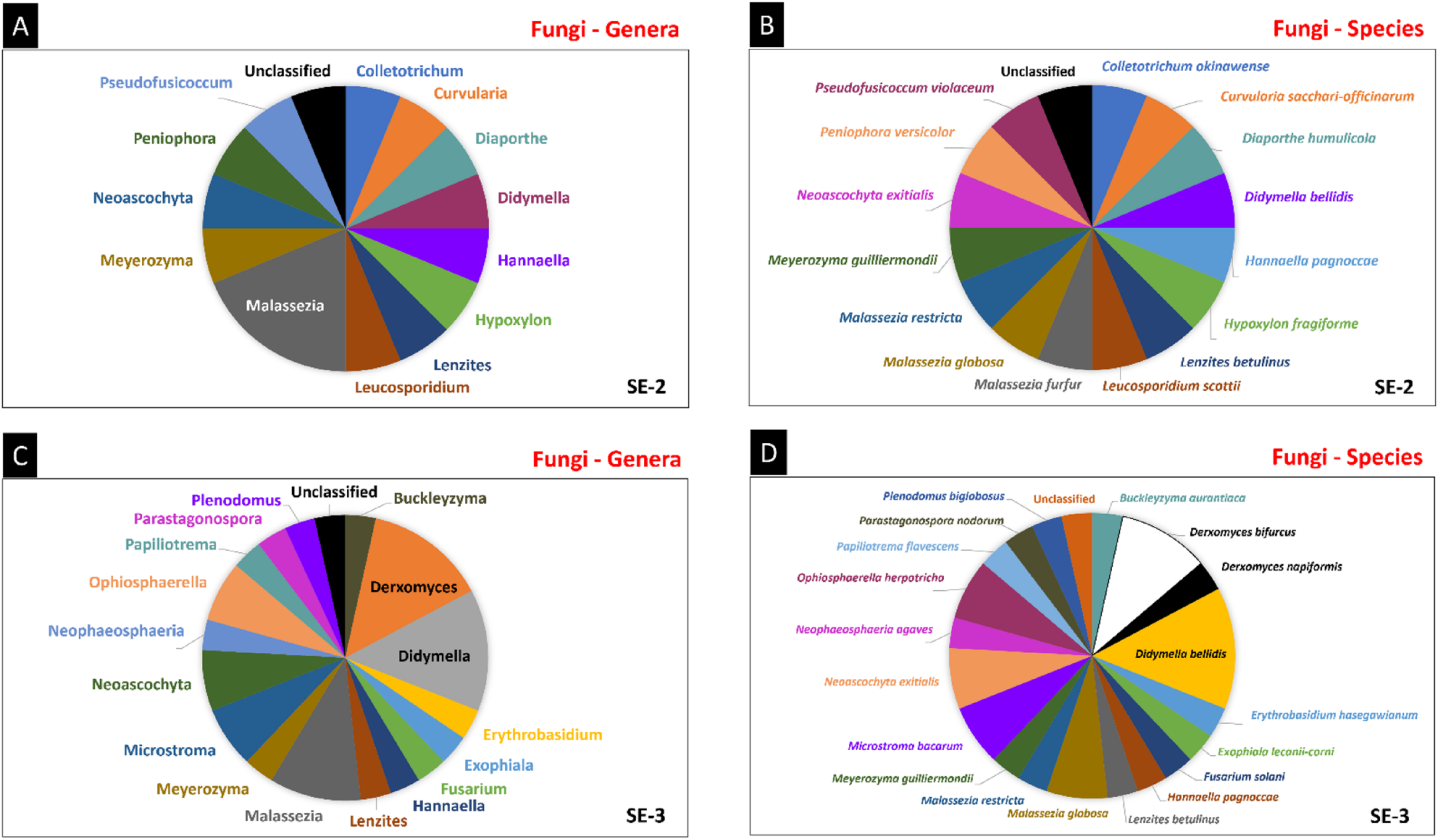
Metagenomic study of the endophytic colonization of *S. erecta* leaves. The three samples (SE-1, SE-2 and SE-3) were analyzed for the relative frequency of sequences attributed to fungal genera and species in SE-2 (**A**, **B**) and SE-3 (**C**, **D**).

Most sequences in SE-2 were of the genus *Malassezia* (18.78%) (Fig. 9A), the only one in this sample represented by more than one species, including *Malassezia furfur, Malassezia globosa,* and *Malassezia restricta* (6.26% each) (Fig. 9B). Most sequences in SE-1 were assigned to the genera *Derxomyces* (14.29%), *Didymella* (14.29%), and *Malassezia* (10.71%) (Fig. 9C). *Derxomyces* sequences included *Derxomyces bifurcus* (10.64%) and *Derxomyces napiformis* (3.65%). All *Didymella* sequences were classified as *Didymella bellidis* (14.29%). Malassezia sequences included the sequences of the species *Malassezia globosa* (7.15%) and *Malassezia restricta* (3.56%) (Fig. 9D).

The genus *Malassezia* was found in the three samples analyzed, represented by the species *Malassezia globosa* and *Malassezia restricta*. The genera *Leucosporidium* and *Peniophora* were found in SE-1 and SE-2, with sequences classified as *Leucosporidium scottii* and *Leucosporidium* sp. in SE-1 and as *Leucosporidium scottii* in SE-2. The genus *Peniophora* was represented in these two samples by the species *Peniophora versicolor*. SE-2 and SE-3 also had species from the genera *Didymella, Lenzites, Meyerozyma, Neoascochyta*, and *Hannaella*. The genus *Didymella* was represented by *Didymella bellidis* in SE-2 and SE-3, and *Lenzites* sequences included those of the species *Lenzites betulinus.* The genera *Meyerozyma, Neoascochyta*, and *Hannaella* were represented by the species *Meyerozyma guilliermondii, Neoascochyta exitialis,* and *Hannaella pagnoccae,* respectively, in these two samples.

## Discussion

### Epiphytic fungi interact with *S. erecta* leaf tissues, penetrating through the stomata or the base of the trichomes and trapping nematodes

Some studies showed that the internal colonization of plant tissues begins with the branching of hypha and penetration through the epidermis^32^. Evidence of epidermal penetration and prevalence of endophytic fungi in *S. erecta* leaf tissues indicates the ability of these fungi to survive the effects of metabolites and resistance mechanisms of this plant. Cell or spore transmission through stomata is one of the main routes by which microorganisms access interior plant systems^33,34^. We observed hyphal penetration through open stomata. The results showed symbiotic relationships between *S. erecta* and leaf surface epiphytic microorganisms since stomatal immunity against fungal invasion includes chitin-induced stomatal closure^35^. A study by Kumar et al.^36^ showed that *Colletotrichum gloeosporioides* germ tubes and its hyphae were oriented toward open stomata, avoiding closed stomata, with the hyphae entering the leaves through stoma openings. Some studies showed that fungal chitinases can convert chitin into chitosan, a compound that does not interact with immune response-inducing receptors, to counteract stomatal immunity^37,38^.

Trichome penetration may be another mechanism for the horizontal transmission of fungi in *S. erecta.* A substantial accumulation of hyphae was evident in the proximity of these structures, with concurrent penetration into their interior. Marques et al.^39^ also demonstrated that fungal hyphae can use the base of glandular trichomes to penetrate leaf tissues. Data shows that fungal hyphae can use the base, side, or top of trichomes for colonization, spreading to adjacent cells^40^. Trichome penetration can be asymptomatic or mildly symptomatic^38^, triggering symbiotic processes. Wang et al.^41^ showed that *Rosa roxburghii* exhibit glandular trichome cells with thin cell walls and plasmodesmata with large intercellular spaces. On the other hand, the outer wall of these trichomes seems to exhibit a thinner configuration at the base, as reported by Argyropoulou et al.^42^, who analyzed secretory trichomes in *Lippia citriodora*. This may help understand the high concentration of epiphytic hyphae associated with the base of *S. erecta* leaf glandular trichomes.

We also visualized fungi physically trapping nematodes for parasitism or predation on the epidermal surface of *S. erecta.* Filamentous fungi capable of capturing free-living nematodes from capture structures are known as nematode-trapping fungi^43^. According to Jones^44^, hyphae-hyphae fusions adhered to nematodes form three-dimensional traps. Conversely, similar to hyphae, spores also adapt to adhere to pathogens by secreting adhesive compounds^44^. These compounds help fungi capture live nematodes and their eggs. The corresponding process involves several steps: attraction, recognition, trap formation, adhesion, penetration, and digestion^45^. Nematodes are attracted to mechanical (constrictor rings) or adhesive fungal traps (adhesive nets, adhesive buttons, adhesive columns, non-constrictor rings) where they are trapped or activate fungal capture mechanisms (restriction traps). The nematode is then paralyzed and the hyphae pierce its cuticle, growing inside the nematode body and completely degrading it by eliminating digestive enzymes^45,46^.

### The cultivable endophytic microbiota of *S. erecta* leaves include phytopathogenic fungi

Electron micrographs showed endophytic fungi in *S. erecta* leaf tissues. Previous studies have also shown biotrophic interactions involving leaf endophytic fungi in the epidermis of this plant, particularly *Bipolaris* and *Curvularia* species^31^. The literature reports the isolation of endophytic fungi from *S. erecta* due to their phytopathogenic habit; however, these fungi express symbiotic traits when associated with *S. erecta*. Endophytic fungi positively affect host plants by producing phytohormones, improving nutrient acquisition, and protecting against invading pathogens, either via direct antibiosis or activation of induced resistance mechanisms in the plant^47,48^.

The species *Colletotrichum gigasporum, Diaporthe schini, Lasiodiplodia theobromae, Macrophomina pseudophaseolina, Nigrospora sphaerica*, and *Pseudofusicoccum* sp. were isolated from *S. erecta* leaf tissues. Silva et al.^49^ suggested the genus *Colletotrichum* has different lifestyles, including phytopathogenic, endophytic, and hemibiotrophic fungi. Species of the genus *Colletotrichum* have already been isolated as endophytes from various plants, including *Nothapodytes pittosporoides, Morus alba,* and *Dendrobium* spp.^50–52^. The species *Colletotrichum gigasporum* appears to be widely distributed in tropical regions, being isolated from *Centella asiatica, Stylosanthes guianensis,* and *Coffea arabica*^53^.

The species *Diaporthe schini* may have phytotoxic activity. Brun et al.^54^ demonstrated that a bio-herbicide produced from *D. schini* metabolites effectively suppressed the growth of *Bidens pilosa L., Amaranthus viridis L., Echinochloa crusgalli* (L.) Beauv., and *Lolium multiflorum* Lam. weeds. In addition, the data presented by Zhou et al.^55^ showed that strains of the genus *Diaporthe* consistently reduced gall formation by nematodes. The nematicidal behavior of this genus corroborates the presence of nematode-trapping fungi on the leaf surface of *S. erecta*.

*Lasiodiplodia theobromae* is a fungal species recognized for its pathogenicity, leading to the demise of tropical fruit trees in the northeastern region of Brazil. When introduced to mango fruits and young cashew, *Annona spp.*, and *Spondias spp.* plants, it induces necrotic lesions of varying severity levels^26,56^. Initially regarded as a pathogen with limited impact, primarily affecting stressed plants, this species has evolved into a prominent pathogenic agent significantly influencing the viability and productivity of tropical fruit trees in Brazil. Its effects encompass stem canker, Leucostoma canker, plant mortality, and postharvest fruit decay^57^. *Lasiodiplodia* can infect a wide range of host plants or survive as saprophytes or endophytes in seeds and other living tissues^58,59^.

Fungi of the genus *Macrophomina* have a wide geographical distribution. They have been described as one of the most destructive phytopathogenic fungi^60^. However, *Macrophomina pseudophaseolina* does not trigger pathogenic symptoms in *S. erecta*. In sensitive plants, this pathogen grows rapidly and produces a large number of sclerotia, which clog the vessels, resulting in plant wilting^61^. A study assessed the nematophagous potential of *M. phaseolina*. The results showed 98% mortality in *Meloidogyne javanica* after 48 h of exposure^62^. Considering that *D. schini* and *M. pseudophaseolina* are a part of the endophytic microbiome of *S. erecta*, we suggested the development of extensive examination methods to discern whether the nematophagous fungi observed by SEM in this study correspond to *D. schini* or *M. pseudophaseolina*.

*Nigrospora sphaerica* leads to the development of rust on *Camellia sinensis* leaves and spots on *Vaccinium corymbosum* leaves, branches, and shoots^63,64^. However, an endophytic *N. sphaerica* isolate obtained from the medicinal plant *Euphorbia hirta* demonstrated the ability to synthesize phenolic compounds and flavonoids^65^. *Pseudofusicoccum* sp. is associated with death, canker, and fruit rot in several tropical hosts, including mango and *Carya illinoinensis*^66,67^. In contrast, endophytic *Pseudofusicoccum* sp. isolates can synthesize phenolic compounds and cyclopeptides^68,69^.

### The metagenomic study of the endophytic colonization of *S. erecta* leaves showed low microbial diversity and a tendency toward a higher number of fungal species

We recovered a total of 90 species from *S. erecta* leaf samples, with 57 ASVs being assigned to bacteria. Other studies with medicinal plant leaves recovered a greater number of OTUs, such as 174 bacterial OTUs in *Aloe vera* and 114 OTUs for endophytic bacteria in *Mentha longifolia*^70,71^. Liu et al.^72^ revealed that the leaf microbiome of *Paris polyphylla* var. *yunnanensis* harbours 910 OTUs. In *Hamamelis virginiana* L., the endophytic microbiome of the leaves totalled 501 bacterial species and 68 fungi, while in *Achillea millefolium* L. these values reached 155 bacteria and 52 fungi respectively^73^, that is, microbiomes more diverse than that found in *S. erecta*. Work with medicinal plants suggests that the Shannon index in the leaf endophytic microbiome is lower than in the root microbiome, given the selective pressure induced by the presence of secondary metabolites in the leaves^72^.

Antimicrobial secondary metabolites in *S. erecta* leaves restrict colonization and reduce the diversity of leaf endophytic microbiome, especially of bacteria. These bioactive compounds include flavonoids, alkaloids, and essential oils^31^. Cardoso et al.^28^ reported that *S. erecta* leaf extract exhibited antibacterial/antifungal activity against *Mycobacterium tuberculosis*, *Staphylococcus aureus*, *Pseudomonas aeruginosa, Salmonella setubal*, *Candida albicans*, *Saccharomyces cerevisiae*, and *Escherichia coli.* The extract also inhibited the growth of *Mycoplasma arginini, M. hominis*, and *Ureaplasma urealyticum*^74^ due to its toxicity^75^. Thus, the endophytic conditions facilitated by *S. erecta* could potentially exert a subtle influence on fungi, rendering them more susceptible to epiphytic (as observed by SEM) and endophytic colonization (as seen in the metagenomic study). On the other hand, fungi that inhabit this plant may be prone to inducing pathways associated with defense responses. Studies attest that endophytes can induce the phenylpropanoid pathway, in which several defense compounds are formed, including pathogenesis-related proteins (PR)^76,77^. Thus, since the endophytic microbiome of medicinal plants can have a significant impact on the production of unique secondary metabolites and pharmacologically active substances^78–80^, we suggest that *S. erecta* endophytic fungi be examined extensively to elucidate the effects of these microorganisms on the potential of this species to produce beneficial bioactive compounds.

### The metagenomic study of the endophytic colonization of *S. erecta* leaves showed association between the nematodes of the genus halicephalobus with leaf tissues, the presence of methanogenic archaea, and a predominance of Alphaproteobacteria among the sequences

The classification of *Halicephalobus* sp. sequences corroborated the presence of nematode eggs on the epidermal surface of *S. erecta,* as observed by SEM. This genus exhibits an almost cosmopolitan distribution^81^, with studies showing insects as vectors for the entry of *Halicephalobus nematodes* into fungal colonies^82^ or plant tissues^83^. However, the exclusive presence of methanogenic archaea may indicate the presence of organisms reducing the availability of CO_2_ in *S. erecta* tissues for methane^84^. This process would constitute a mechanism of metabolic competition with *S. erecta*, but the physiological benefits of these archaea should symbiotically balance the cost of maintaining them. An analysis of the rhizospheric and root endophytic microbiome of *Oryza longistaminata* identified a great diversity of methanogenic archaea^85^.

The CH_4_ generated by these archaea may be conveyed through *S. erecta* leaf tissues, potentially creating a microenvironment rich in both inorganic and organic substances. This environment, inclusive of C1 compounds like methane, could support the proliferation of methanotrophs utilizing CH_4_ and methanol as carbon sources^86,87^. Thus, a considerable number of methanotrophs such as *Methylobacterium* can develop in the tissues. It is key to maintain Alphaproteobacteria of the genus *Methylobacterium* in plant tissues because they can fix atmospheric nitrogen and produce the hormone cytokinin and the enzymes pectinase and cellulase, thus increasing plant growth due to nitrogen availability and systemic resistance induction^88–90^. Bacteria of the genus *Methylobacterium* were found in *S. erecta* SE-1 and SE-3.

Associated with this, the Alphaproteobacteria *Atribacter laminatus* present in SE-1 and SE-2 belongs to the phylum *Atribacterota*, which commonly has species in anoxic sediments rich in methane. Genetic analyses suggest a heterotrophic metabolism producing fermentation products such as acetate, ethanol, and CO_2_. These products support methanogenic substances within the microbial community, explaining their co-occurrence with methanogenic archaea^91,92^. Alphaproteobacteria of the genus *Sphingomonas* were also found in SE-1 and SE-3 sequences. These bacteria have been described as common and abundant in plant tissues, being found in the microbiome of 26 plant species in 11 families. The maximum level of *Sphingomonas* was 10^8^ g^−1^ (wet weight) of plant tissue, demonstrating large populations^93^. Some studies showed that species in this genus improve plant growth under stressful conditions such as drought, salinity, and the presence of heavy metals in agricultural soils. This role has been attributed to its potential to produce plant growth hormones such as gibberellins and indole-acetic acid^94^.

Metagenomic studies support the idea that the microbiome residing in medicinal plants may be considerably variable between species^95^. Our work corroborates these studies, as we observed in the leaf microbiome of *S. erecta*, a generic composition different from that observed, for example, in *Aloe vera*, where the genera *Pseudomonas* and *Bacillus* predominated^96^. *Cyanobacteria* and *Rhizobium* were the most frequent genera in the leaf microbiome of *Paris polyphylla* var. *yunnanensis*. Here, *Rizobium* appears at low frequency, and only in SE-3^72^. *Streptophyta* was the dominant genus found in *Senna italica* leaf samples^97^. In the leaves of *Bouvardia ternifolia*, the predominant genera were *Erwinia*, *Propionibacterium* and *Microbacterium*, genera that did not appear in the *S. erecta* samples^98^. In the case of *Dicoma anomala*, *Cutibacterium* sequences were predominant in the leaves, but also the genera *Acinetobacter* and *Methylobacterium*^99^, observed at high frequency in *S. erecta*.

### The metagenomic study of endophytic fungi in *S. erecta* showed a predominance of the phylum ascomycota, mainly comprising plant growth-promoting yeasts

Ascomycota species form symbiotic relationships with plants, with important functions such as nutrient acquisition and disease suppression^100,101^. In this study, the three samples of *S. erecta* analyzed had fungi of the genus *Malassezia,* including the species *Malassezia globosa* and *Malassezia restricta*. *Malassezia* was also one of the most frequent genera in the microbiome of stigmas and pistils in *Orobanche* *alsaticae* flowers^102^, with *Malassezia restricta* being a very common species. Elhady et al.^103^ showed that *Malassezia globosa* was the most frequent species in the microbiome of nematode-suppressive agricultural soil. *Malassezia restricta* and *Malassezia globosa* were found in the leaf microbiome of *Astragalus canadenses* and in several developmental stages of the butterfly *Lycaeides melissa*, which hosts *A. canadenses*^104^.

*S. erecta* leaves showed a tendency to accumulate yeasts in their endophytic microbiome, especially of the genera *Malassezia, Leucosporidium, Meyerozyma,* and *Hannaella*. The genera *Leucosporidium* was also sampled as part of the leaf microbiome of pasture plants^105^, but studies have shown that these yeasts may also be present in the phyllosphere microbiome^106^. *Meyerozyma* is considered a yeast that promotes plant growth^107^, being observed in the endophytic microbiome of grains^108^ and also in fruits of plants in the Cerrado biome^109^. The species *Meyerozyma guilliermondii* is promising in reducing the effects of abiotic stress, increasing tolerance, and improving crop performance^110,111^. This species is well known for its antagonistic effects against phytopathogens such as *Fusarium equiseti*^112^. The genus *Peniophora* was present in SE-1 and SE-2. Wu et al.^113^ suggested that species of this genus promote plant growth. *Peniophora* and *Lenzites* synthesize laccases, which are important enzymes that act in plant morphogenesis, fungal plant-pathogen/host interaction, defense against stress, pigment formation, and phenolic compound detoxification^114–116^. The genus *Hannaella* and the bacteria *Sphingomonas* and *Methylobacterium* are the most abundant in the seed microbiome of six different *Oryza sativa* genotypes, consistent with the findings of the present^117^.

We suggest further studies with *S. erecta* to identify other microbiomes, such as rhizospheric and root, and the leaf endophytic microbiome of other plant species. This will help us understand the effective role of the microorganisms found in this study in the microbiomes of different plant species, especially in medicinal plants such as *S. erecta*. However, none of the endophytic species isolated from *S. erecta* leaf tissues were identified in metagenomic sequences, indicating that isolation and metagenomic techniques are not mutually exclusive, contributing to a comprehensive understanding of microbial diversity found in the microbiome of medicinal plants.

## Conclusions

In the presented study it was depicted that epiphytic fungi interact with *S. erecta* leaf tissues. They are horizontally transmitted to internal tissues via stomata or the base of the trichomes and express functional traits for trapping nematodes. Cultivable endophytic fungi isolated from *S. erecta* are known for their phytopathogenic habits; nevertheless, their association with *S. erecta* did not elicit dysbiosis symptoms. We confirmed the hypothesis of low microbiome diversity in *S. erecta,* with a tendency toward a higher number of fungal species, suggesting that this is an effect of antibacterial secondary metabolites present in the leaves. In contrast, the classification of *Halicephalobus* sp. sequences corroborated the presence of nematode eggs on the epidermal surface of *S. erecta*. In addition, we confirmed the presence of methanogenic archaea and a considerable number of methanotrophs of the genus *Methylobacterium*. The methanogenic study of endophytic fungi showed plant growth-promoting yeasts, mainly of the genera *Malassezia, Leucosporidium, Meyerozyma*, and *Hannaella*. Endophytic fungi and other microbiomes isolated from *S. erecta* should be examined to understand the effects of these microorganisms on the capacity of the species to produce beneficial bioactive compounds.

## Materials and methods

### Collection of biological materials

**Figure 10:**
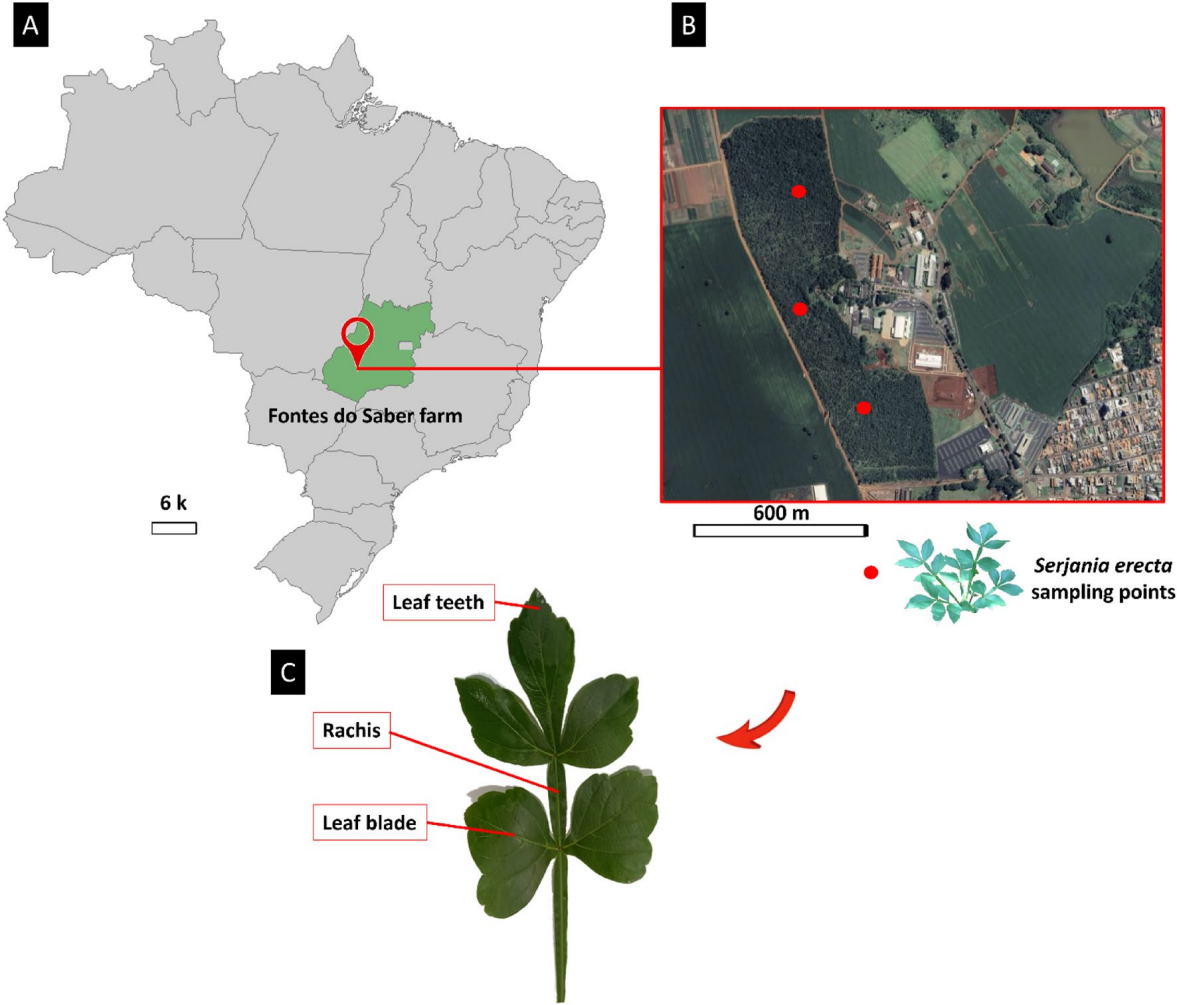
Sampling area of *S. erecta* samples (SE-1, SE-2 and SE-3) at Fontes do Saber farm, Rio Verde, GO, Brazil (**A**), Sampling points in the experimental area, (**B**) and location of the different leaf regions evaluated: rachis, teeth, and blade (**C**). Map constructed using ArcGIS Pro 3.1.5 (ESRI) software, obtained from https://www.esri.com/en-us/arcgis/products/arcgis-pro.

Healthy leaves were sampled from three *S. erecta* samples (SE-1, SE-2 and SE-3) collected in a field at Fontes do Saber farm, Rio Verde, GO, Brazil (-17.783262° S -50.967928° W), an area of transitional vegetation between strict Cerrado and Cerradão (Fig. 10A,B). The collection of adult shrub-like samples was previously authorized by the SISBio (Biodiversity Authorization and Information System) under license no. 92592/1 and by the Instituto Federal Goiano under registration no. 011/2021. Thus, all necessary licenses and permissions were obtained for the development of the study, and the plant collection and use was in accordance with all the relevant guidelines. The samples were carefully packed in previously sterilized plastic bags and stored in thermal boxes containing ice. The material was immediately sent to the Laboratory of Agricultural Microbiology, Instituto Federal Goiano, Rio Verde campus, for processing. The leaves were carefully sectioned from the stem using a sterilized scalpel and sent for analysis of leaf surface colonization by epiphytic fungi, internal leaf tissue colonization by endophytes, and metagenomic study. A voucher sample was deposited for identification confirmation at the herbarium of the Instituto Federal Goiano, Rio Verde campus with a catalog number of 545. The material was then sent to Dr. Germano Guarim Neto at the Federal University of Mato Grosso for identification. The identification of the material as *Serjania erecta* Radlk was confirmed.

Epidermal surface epiphytic colonization was assessed by SEM, and endophytic colonization in internal leaf tissues was evaluated by isolation in culture medium. Metagenomic analyses with next-generation sequencing were performed to determine the resident endophytic microbiome.

### Scanning electron microscopy

Leaf fragments of approximately 3 mm^2^ were obtained from the rachis, blade, and teeth of *S. erecta* leaves (Fig. 10C). The fragments were fixed in formalin, acetic acid, and 70% ethyl alcohol and analyzed at the SEM laboratory of the Regional Center for Technological Development and Innovation (CRTI) (for details on *S. erecta* leaf anatomy, see Freitas^31^). The samples underwent a critical point drying process, compositional analyses, and coating with gold, which served as a conductive element for image acquisition.

Images from the epidermal surface of the three samples were captured using a Jeol JSM7100F field emission SEM microscope (SEM-FEG) with an electron acceleration voltage of 5 keV in secondary electron detection (SED) mode. Alternatively, images from longitudinal and transverse sections were used to assess the interaction of hyphae and fungal structures with *S. erecta* internal leaf tissues.

### Isolation of endophytic fungi

Endophytic fungi were isolated following the methodology proposed by dos Reis et al.^118^. Thus, the *S. erecta* leaf samples were superficially disinfected to eliminate epiphytic microorganisms. They were then successively rinsed in ethanol (70%), sodium hypochlorite 2.5% (active chlorine), and ethanol (70%) solutions for 1 min, 5 min, and 30 s, respectively. At the end of the process, the samples were rinsed four times in autoclaved distilled water, and a 100 µL aliquot was collected during the last rinse for inoculation in nutrient broth (3 g meat extract, 5 g peptone) at 28 °C for 24 h to test the efficiency of the disinfestation process.

Aseptic leaf fragments of approximately 1 cm^2^ (1 fragment per leaf region, analyzed in triplicate per sample, totaling 9 fragments per sample) were placed in Petri dishes containing potato dextrose agar (PDA) medium (potato infusion broth, 200 ml; dextrose, 20 g; agar, 17 g; q.s.p. 1,000 ml, final pH = 5.6 ± 0.2) supplemented with azithromycin (500 mg L^−1^). The plates were incubated at 30 °C and monitored for seven days. Fungal colonies possibly associated with leaf tissues were purified by removing mycelial fragments using an inoculation loop and transferring them to plates containing PDA.

### Molecular identification of fungal isolates

For molecular identification, fungal isolates were grown in potato dextrose (PD) broth (potato infusion broth, 400 ml; dextrose, 20 g; q.s.p. 1,000 ml, final pH = 6.6 ± 0.2) for seven days. Subsequently, genomic DNA was extracted in triplicate from each isolate, following the method proposed by Cheng and Jiang^119^ and using a Minirep extraction kit (Axygen biosciences, USA) according to the manufacturer’s instructions. Identification was carried out by sequencing the ITS-1 and β-tubulin regions after amplification and purification. The Sanger method was used for sequencing, and the sequences were paired by similarity with sequences from the GenBank for phylogenetic inference using the nucleotide Basic Local Alignment Search Tool (BLASTn)^120^ considering homology greater than 98%. The fungal sequences obtained were concatenated and aligned with the sequences of nine fungal species, also obtained from the GenBank (*Colletotrichum gigasporum, Diaporthe schini, Lasiodiplodia theobromae, Pseudofusicoccum adansoniae, Pseudofusicoccum violaceum, Pseudofusicoccum stromaticum, Macrophomina pseudophaseolina, Macrophomina phaseolina, and Nigrospora sphaerica*). The sequences were aligned using the Clustal Omega software^121^.

The sequence evolution model was selected according to the Bayesian Information Criterion (BIC) using the jModelTest 2 software^122^. The selected model was HKY + G with a gamma shape of 1.8010. Phylogenetic trees were independently inferred for bacteria and fungi using Bayesian inference methods in the MrBayes v.3.2.6. software^123^. Each tree underwent four independent runs, with 10 × 10^6^ generations assigned to the chains and a posteriori probability distribution obtained every 500 generations. The first 2,500 trees sampled were discarded before calculating the consensus trees, one for bacteria and one for fungi, to ensure chain convergence. The phylogenetic tree with the highest Bayesian probability was visualized and edited using FigTree v 1.4.4^124^.

### Genetic data for metagenomic analysis

The community of total endophytic fungi was assessed using *S. erecta* leaves previously subjected to the disinfestation process described above, ensuring total epiphyte removal. The leaves were then placed in liquid nitrogen until analysis. DNA was extracted using the PowerSoil Pro Kit (QIAgen), from 200 mg of leaf tissue per replicate, each sample (SE-1, SE-2 and SE-3) was analyzed in triplicate. DNA concentration and purity were monitored by electrophoresis on 1.0% (w/v) agarose gel. V3-341F (CCTACGGGNGGCWGCAG) and V4-785R (GACTACHVGGGTATCTAATCC) primers^125^ were used for amplification of the 16S rRNA gene region of bacteria and archaea, Euk1391F (GTACACACCGCCCGTC) and EukBR (TGACCTTCTGCAGGTTCACCTAC) were used to amplify the 18S rRNA^126^ gene region of the fungi, and the amplification products were visualized on 1.5% (w/v) agarose gel. After quantification, qualification, grouping, and purification, polymerase chain reaction (PCR) amplification products were sequenced using the KAPA Library Quantification kit for Illumina (Roche) on an Illumina MiSeq sequencer with a coverage of 300,000 reads per sample.

### Bioinformatics analysis

We utilized the Nextflow pipeline ampliseq v.2.7.1 (available at [https://github.com/nf-core/ampliseq]) for the analysis of 16S and 18S rRNA sequencing data. Initially, the integrity of raw sequencing reads was assessed using FastQC^127^, followed by the removal of primer sequences using cutadapt v.2.7^128^. Subsequent processing involved denoising, dereplication, and chimeric sequence filtration through DADA2^129^. The denoised paired-end reads were truncated from position 233 (forward) and 229 (reverse) after a thorough manual inspection of the sequencing error patterns. Reads that did not meet specific length criteria were excluded. The truncated sequences were merged with at least a 20 bp overlap, resulting in exact amplicon sequence variants (ASVs). These ASVs were taxonomically classified from phylum to species level and clustered with 99% similarity using the SILVA v132 database^130^. Following the taxonomic classification of ASVs into OTUs (Operational Taxonomic Units) by applying a Naïve Bayes classifier implemented in QIIME2^131^ trained on the preprocessed database. Subsequently, ASVs associated with mitochondrial or chloroplastic origins were filtered. For downstream analysis, we exclusively considered ASVs with a read frequency of ≥ 5 in at least one sample. Furthermore, rarefaction curves were employed to evaluate whether the sequencing depth adequately captured the full extent of species richness within the samples.

### Supplementary Information

Supplementary Information.

## Data Availability

Data may be made available by contacting the corresponding author.
