# Supplementary material for: Low microbial diversity, yeast prevalence, and nematode-trapping fungal presence in fungal colonization and leaf microbiome of Serjania erecta
Source: Sci Rep. 2024 Jul 4;14:15456. doi: 10.1038/s41598-024-66161-3 (PMC11224404; doi:10.1038/s41598-024-66161-3)
Supplement: Supplementary file 1 — Supplementary Information. [file 41598_2024_66161_MOESM1_ESM.docx]

**Low microbial diversity, prevalence of yeasts, and presence of nematode-trapping fungi in the analysis of fungal colonization and leaf microbiome in the medicinal plant *Serjania erecta***

**Table 1S.** Fungal species isolated as foliar endophytes of *Serjania erecta* and the respective accession numbers for molecular data in Genbank.

| **Species** | **Genbank access numbers** |
| --- | --- |
| *Colletotrichum gigasporum* | PP780052 |
| *Diaporthe schini* | PP784309 |
| *Lasiodiplodia theobromae* | PP784960, PP784961, PP784962, PP784963 |
| *Macrophomina pseudophaseolina* | PP784964 |
| *Nigrospora sphaerica* | PP784306 |
| *Pseudofusicoccum* sp. | PP784307 |


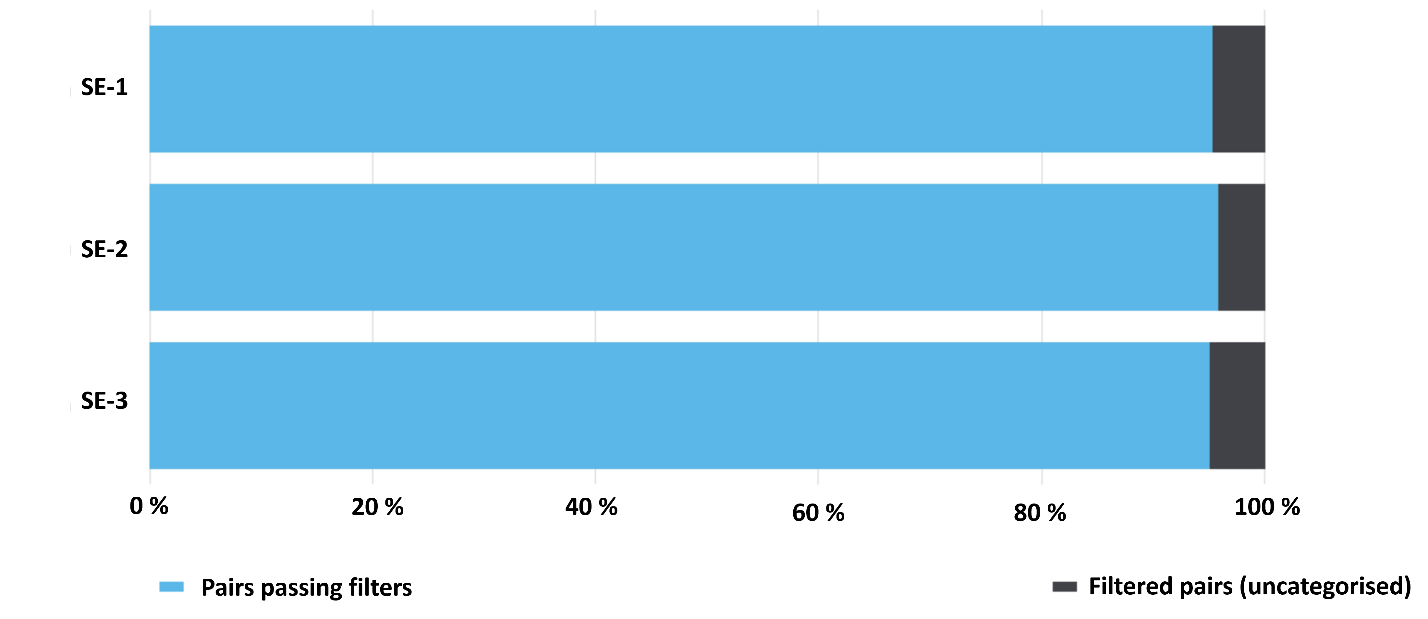


**Figure 1S.** Percentage of reads used and eliminated by the filter in the metagenomic analysis of *S. erecta* leaf endophytic colonization. Three samples (SE-1, SE-2 and SE-3) were analyzed.


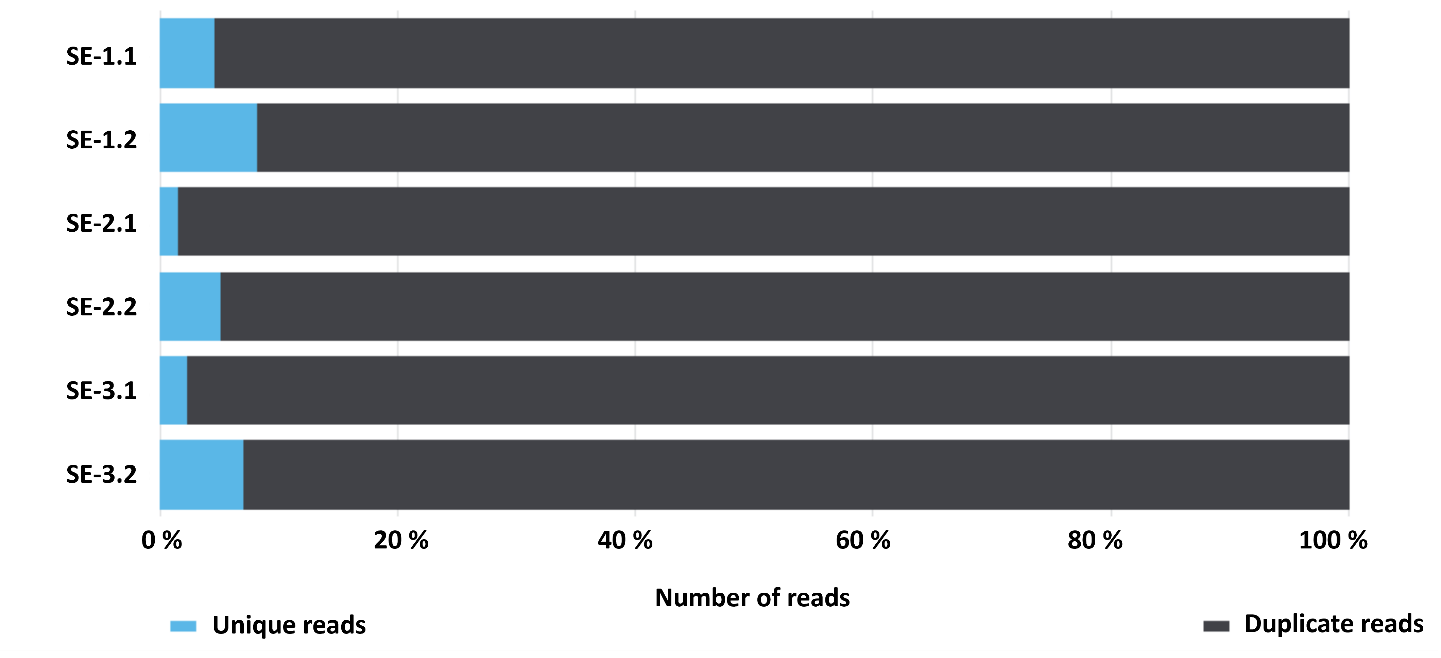


**Figure 2S.** Percentage of unique and duplicate reads obtained in the metagenomic analysis of *S. erecta* leaf endophytic colonization. Three samples (SE-1, SE-2 and SE-3) were analyzed.


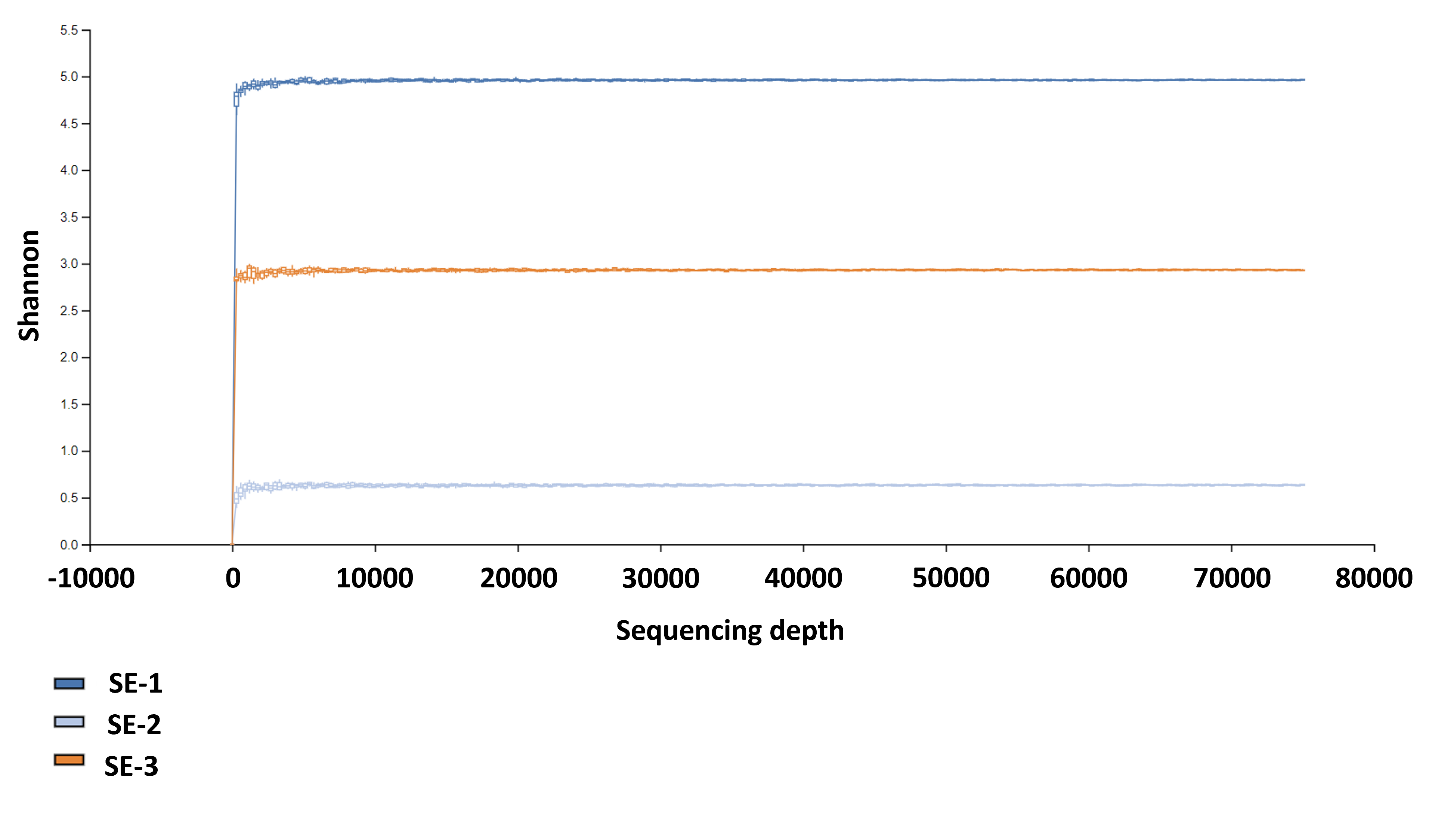


**Figure 3S.** Shannon diversity index levels in the metagenomic sequencing of *S. erecta* leaf endophytic colonization. Three samples (SE-1, SE-2 and SE-3) were analyzed.

**Table 2S.** Evidence for identification of the nematode *Halicephalobus* sp. (class Chromadorea), present in SE-2 and SE-3.

| **Sequence** | **Sample** | **Relative frequency** | **Identity to *Halicephalobus* sp. (%)** | **Closest access** |
| --- | --- | --- | --- | --- |
| GCTGCCTAGAACTGAGTTGATTCGAGAAGATGGGGGAGAGTAGTATATGTAGCAATGCATATATTGTTCGAACCCATTTGATCGTATTGGCCTGAACCAGGCAAAAGTCGTAACAAGGTGGCC | SE-2 | 3.45 | 98.37 | MK087057.1 |
| GCTGCCTAGAACTGAGTTGATTCGAGAAGATGGGGGAGAGTAGTATAAGTAGCAATACTTATATTGTTTGAACCCATTTGATCGTATTGGCTTGAACCAGGCAAAAGTCGTAACAAGGTGGCC | SE-3 | 1.79 | 97.56 | MK087060.1 |
